# Initial development and testing of a novel foam-based pressure sensor for wearable sensing

**DOI:** 10.1186/1743-0003-2-4

**Published:** 2005-03-01

**Authors:** Lucy E Dunne, Sarah Brady, Barry Smyth, Dermot Diamond

**Affiliations:** 1 Adaptive Information Cluster, Department of Computer Science, University College Dublin, Belfield, Dublin 4, Ireland; 2 Adaptive Information Cluster, National Centre for Sensor Research, Dublin City University, Dublin 9, Ireland

## Abstract

**Background:**

This paper provides an overview of initial research conducted in the development of pressure-sensitive foam and its application in wearable sensing. The foam sensor is composed of polypyrrole-coated polyurethane foam, which exhibits a piezo-resistive reaction when exposed to electrical current. The use of this polymer-coated foam is attractive for wearable sensing due to the sensor's retention of desirable mechanical properties similar to those exhibited by textile structures.

**Methods:**

The development of the foam sensor is described, as well as the development of a prototype sensing garment with sensors in several areas on the torso to measure breathing, shoulder movement, neck movement, and scapula pressure. Sensor properties were characterized, and data from pilot tests was examined visually.

**Results:**

The foam exhibits a positive linear conductance response to increased pressure. Torso tests show that it responds in a predictable and measurable manner to breathing, shoulder movement, neck movement, and scapula pressure.

**Conclusion:**

The polypyrrole foam shows considerable promise as a sensor for medical, wearable, and ubiquitous computing applications. Further investigation of the foam's consistency of response, durability over time, and specificity of response is necessary.

## Background

We live in a world of information, and emerging technologies compel us to look for new ways to collect, process, and distribute information. Today we are faced with a significant information overload problem as users struggle to locate the right information in the right way at the right time. In response, a number of researchers have suggested that adaptive information technologies may hold the key to the next generation of ubiquitous information systems, systems that automatically adapt to changes in their environment and usage in order to deliver users a more intelligent, proactive and personalized information service. In this paper we provide an overview of initial research conducted as part of the Adaptive Information Cluster  a multi-disciplinary research cluster that brings together researchers in areas such as wearable computing, sensor technologies, information retrieval and artificial intelligence with a view to developing the next generation of intelligent, sensor-based wearable computing technologies.

Sensing in the wearable environment is crucial for many applications, but existing sensor technologies pose significant wearability problems when integrated into the user's peri-personal space. One of the most compelling needs for wearable technology is in the continuous monitoring of the human body, be that for medical monitoring or to inform the operation of a context-aware computerized application. While many technologies that are often made wearable (such as music players or telephones) function nearly as well (or sometimes better) as portable devices, almost all continuous body-sensing technologies must be worn to be effective. However, because of their ubiquitous, constant-wear nature, such technologies must prioritise the effects of the technology on the user's physical comfort as well as social comfort. Traditional sensing technologies are rarely designed for continuous, on-body use: those that require skin contact are generally designed to be used in a hospital or doctor's office, and those that do not are generally designed for use in stationary devices. Consequently, the achievement of certain design goals for existing sensors (such as durability) is ultimately detrimental to the user's comfort when applied to the wearable environment. For example, durability often equals stiffness, which results in a solid device that can cause discomfort by localizing pressure.

Textile-based sensors offer a compromise solution to this problem, by retaining the characteristics associated with comfort and wearability (properties of standard, non-electronic garments). Many textile-based sensors are actually sensing materials used to coat a textile [1] or sensing materials formed into fibres and woven or knitted into a textile structure [2]. The properties sought by textile-based sensors can include flexibility, surface area, washability, stretch, and hand (texture of textile). However, they must also include the properties required for the electronic device, including durability, power consumption, and ease of connection into a circuit. Metallic components, designed to function in rigid environments, often do not satisfy these needs. For instance, a metallic element in a high-flex environment (such as a garment) will soon break. However the recently discovered [3] conducting electroactive polymers (CEP), offer a potential solution to this problem. CEPs such as polypyrrole (PPy), polyaniline and polythiophene constitute a class of polymeric materials which are inherently able to conduct charge through their polymeric structure. They can be reversibly switched from the doped conducting state to the undoped insulating state upon chemical or electrochemical treatment. In particular, polypyrrole has attracted much interest because it is easily prepared as films, powders and composites, has a relatively high conductivity and is relatively stable in the conducting state. However, when the black precipitate of PPy has been formed it is insoluble to all known solvents and is non-processable. To overcome this PPy can be simultaneously polymerised and deposited onto the substrate [3]. The result is that the substrate is covered with a thin layer of PPy rendering the whole object conducting without compromising the mechanical properties of the substrate.

## Methods

### Sensor Development

In previous work [4], a novel polymer synthesis methodology was developed to create a textile-like structure capable of sensing changes in planar or perpendicular pressure, by coating an open-cell polyurethane (PU) foam with a CEP (polypyrrole). The method used for sensor fabrication is described in [4]. The method involved soaking the substrate, the PU foam in an aqueous monomer and dopant solution. An aqueous oxidant solution was then introduced into the reaction vessel to initiate polymerisation. This lead to the precipitation of doped PPy, which subsequently deposited onto the PU substrate.

### Sensor Characterization

Characterisation for the PPy-coated PU foam was carried out using a number of methods as described in [4]. It was found that increasing the weight placed upon the PPy-PU foam or shortening the overall length of the foam resulted in a proportional decrease in the electrical resistance measured across the foam in a linear fashion. Results from tests carried out using the Instron™ tensile testing instrument, courtesy of the University of Bath, England, showed that the stress-strain profile of the unadulterated PU foam sample and that of the PPy-coated PU foams sample were similar showing regions of elastic and inelastic responses to force. Problems such as repeatability and long-term aging of the foam were identified. The issue of repeatability was due to hysteresis effects observed during the tensile testing of the foam. These effects were observed for the coated and uncoated samples thus originating from the PU substrate. The effect of the PPy coating was to make the entire foam conducting without compromising the soft, compressible mechanical properties of the foam substrate.

### Torso Garment

**Figure 1:**
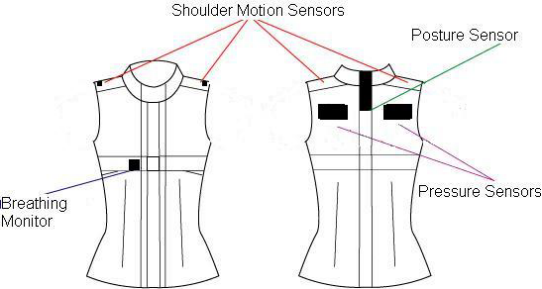
Garment Structure and Sensor Layout

Once a predictable reaction was observed from the foam, it was applied to the wearable environment to explore its utility in garment systems. It was integrated into a torso garment in several ways to investigate the ability of the foam sensor to monitor specific body changes and physiological signals. The test garment contained foam sensors in 6 locations: the top outer edge of each shoulder, the back of the neck, the superior protrusion of each scapula, and the right side rib cage under the bust (Figure 1). Sensor positions were chosen to test the foam reaction to 4 different actions: breathing, shoulder movement, neck movement, and shoulder-blade pressure.

The test garment was a sleeveless, collared shirt, closely fitted and nonextensile. The outer garment layer was a 100% polyester satin weave, and the inner layer was a 100% acrylic satin weave. The collar was 80% nylon, 20% elastine jersey knit. The structure of the garment was crucial to the quality of data obtained, as its textile composition, design, and fit moderated the amount of force present between the body and the sensors. In this study, the prototype garment was fitted to one test subject, to eliminate inter-subject anthropometric variation.

**Figure 2:**
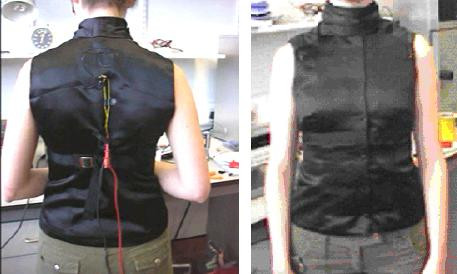
Prototype pressure-sensitive torso garment

Sensors were sewn between the two garment layers, allowing them to be easily removed and interchanged. In each test two wire leads were attached to the foam sensors and to a constant current digital multi-meter, HP, Leixlip, Ireland. Data was collected at a rate of 3 points per second. The finished prototype garment is shown in Figure 2.

#### Breathing

The breathing sensor was attached on the subject's left-side rib cage, under the bust. The sensor measured 2.75 × 1.5 × 0.5 cm. Data was gathered with the subject standing, and the subject was instructed to breathe deeply for a period of approximately one minute.

#### Shoulder Movement

Two shoulder movement sensors were attached at the outer edge of the garment at the apex of each shoulder (above the subject's axilla). The sensors measured 1.5 × 2.0 × 0.5 cm. Data was gathered with the subject seated, and the subject was instructed to raise one shoulder repeatedly to its maximum height.

#### Neck Movement

The neck motion sensor was attached vertically along the subject's spine, at the back of the neck extending from 4 cm below the top of the collar (approximately 2^nd ^vertebra) to 2.5 cm below the neckline of the garment (approximately 4^th ^vertebra). The sensor measured 1.5 × 5.5 × 0.5 cm. Data was gathered with the subject seated, and the subject was instructed to perform four full neck extensions (backwards movement) and three full neck flexions (forward movement).

#### Shoulder Blade Pressure

Two pressure pads were attached, one over the superior edge of each scapula. The sensors measured 8 × 4 × 0.5 cm. Data was collected with the subject alternately supine and seated, on a hard surface.

## Results

### Sensor Characteristics

The sequential coating of PU foam with conducting polymers resulted in an increase of the overall weight of the foam and the conductivity of the foam also from being an insulating material to a conductive material (ca. 1.41 mS/cm). The conductivity of the modified foam depends on the weight of conducting polymer deposited, which in turn depends on the number of coating layers deposited on to the foam substrate. It has been shown previously [4] that by coating the PU foam substrate a total of three times with PPy an electrical resistance of 1 kΩ/cm can be achieved. The PPy-PU foam was rubbed vigorously and rinsed with cold Milli-Q water to remove any loosely bound PPy. The stability of the bound PPy onto the PU substrate was excellent and resistance of the foam did not change with subsequent hand washings in cold Milli-Q water. The electrical conductivity is good remaining in the kΩ/cm region for up to 3 months.

### Torso Garment

Integrating the foam sensors into the torso garment caused little alteration in the visual or tactile properties of the garment. The largest sensors, the scapula pressure pads, caused the only visible change to the appearance of the garment, as these were the only sensors that possessed enough volume to change the surface topology of the garment. Although comfort was not a measured variable, there appeared to be no change in the tactile comfort of the garment when the sensors were added. In demonstration, both the test subject and other viewers had difficulty locating the sensors within the garment without direction.

#### Breathing

**Figure 3:**
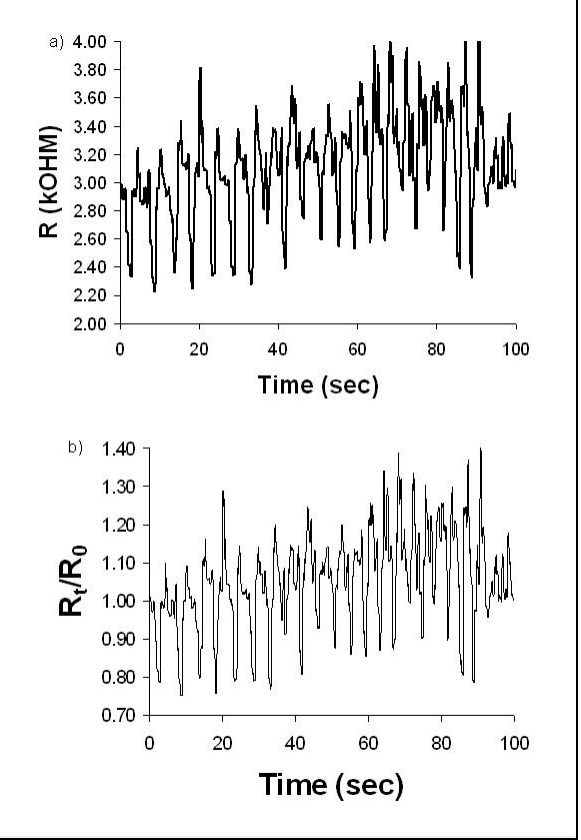
a) Absolute resistance response to Deep Breathing, b) relative resistance response (R_t_/R_0_) to Deep Breathing

As seen in Figure 3a, deep breathing resulted in a sinusoidal resistance curve, varying between approximately 2 kΩ and 4 kΩ. These are absolute values and a low total change compared to the other sensors. This is a result of the age of the foam: The breathing sensor was replaced with week-old foam prior to the test, while the other sensors were 2 months old. The sensor foams are composites of PPy and PU and so the absolute resistance of the foam will be affected by each of these components. Firstly the absolute resistance of the PPy may vary with time due to the gradual oxidation of the polymeric backbone. Also hysteresis in the PU foam substrate as observed during the tensile testing will cause problems to the measured absolute resistance. This hysteresis effect of the PU foam during use can be seen as the gradual and positive drift in the measured resistance that can be seen in Figure 3a. This drift was calculated as 26.67% change of resistance per minute. However if the foam sensor is allowed to relax, un-used, for 2 hours, then the resistance returns to the initial resistance value. However, the sensor output appears to be sufficiently robust, even in its unfiltered state, for a reliable determination of the wearer's respiratory rate, for example. In order to normalise the data so that the sensitivity of the sensor could be determined, the relative resistance of the foam sensor was plotted as in Figure 3b. This was calculated by dividing the absolute resistance at a given time *t*, *R*_*t*_, by the initial baseline resistance, *R*_*0*_. It can be seen in Figure 3b that there was an approximate 20% change in the relative resistance of the foam sensor between inhalation and exhalation.

#### Shoulder Movement

**Figure 4:**
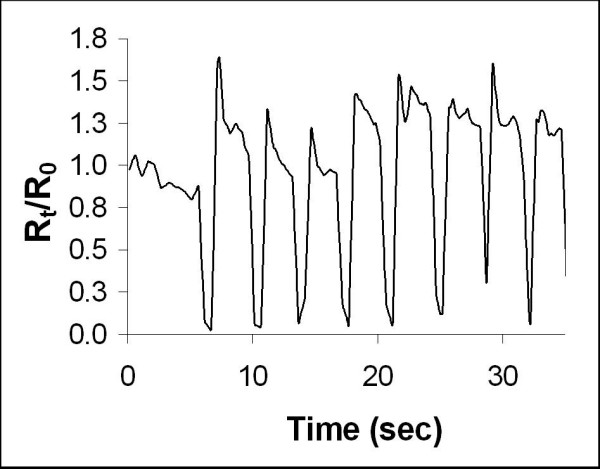
Resistance Response to Shoulder Lift

The response of the foam to shoulder movements was an approximate 100% decrease in relative resistance as seen in Figure 4. Once again the data appears sufficiently robust to reliable detect each shoulder movement; however no test was performed to detect the foam reaction to shoulder movements of varying magnitudes.

#### Neck Movement

**Figure 5:**
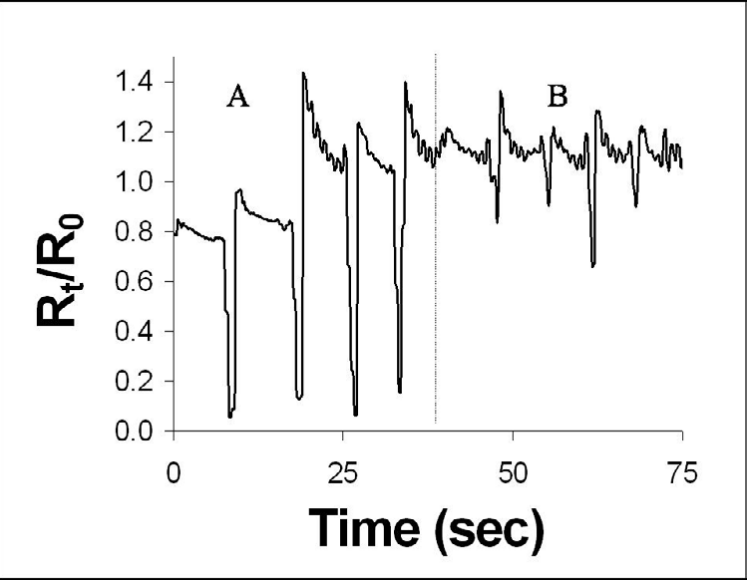
Resistance Response to Neck Movement

The foam responded to full neck extensions, section A in Figure 5, with an 80% decrease in the relative resistance. Full flexion of the neck, section B in Figure 5 involved a smaller body movement, which was detected as a smaller decrease (30%) in the relative resistance of the sensor. This data indicates that the dorsal neck sensor placement exhibits a response of greater magnitude for extension than for flexion. Since the sensor provides no additional qualitative information, it is hypothesized that a second sensor would be required to determine the difference between a small extension and a large flexion.

#### Shoulder-Blade Pressure

**Figure 6:**
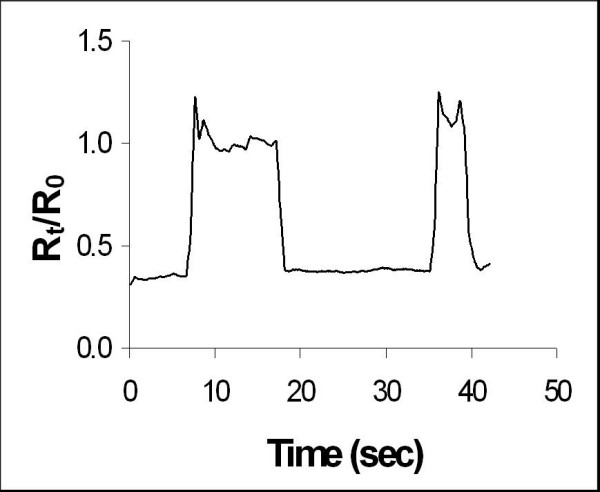
Resistance Response to Constant Scapula Pressure

The foam responded with a 60% increase in the relative resistance when the subject moved from supine (applying pressure to the scapula area) to a seated position (no pressure), as seen in Figure 6. The response time of this sensor, that is, the time taken for the resistance to stabilise after the subject moved to a seated position, was approximately 8 seconds. The response time was shown previously [4] to be inversely related to the force applied to it and is also influenced by the size of the sensor. The foam sensor in this position measured 32 cm^2 ^versus 2–12 cm^2 ^for the other sensors and so the response time for the shoulder-blade foam sensor would be slightly slower than that for the other sensor positions, e.g. 4 seconds for shoulder lift foam sensor.

## Discussion

As demonstrated, pressure sensing in the wearable environment can provide useful descriptive information about the physical state of the user. Conducting electroactive polymers are attractive for sensing in a garment-integrated context because of their ability to retain the tactile and mechanical properties of a textile-based structure. In the garment integration, the foam sensors had little effect on the comfort or wearability of a standard garment. However, more investigation is necessary to determine the accuracy of the foam sensor responses, particularly the repeatability of response.

As seen in the torso sensor evaluation, the age of the sensor had a significant impact on the absolute resistance of the sensors. It has been shown previously that if PPy is left to open to atmosphere then there is a gradual increase in the electrical resistance due to oxidation of the polymeric backbone [5]. However, the coating itself did not delaminate from the foam substrate, even during hand-washing of the foam sensors. This indicates that if the oxidation were prevented, the sensor would be durable and washable over an indefinite period of time. In a garment-integrated context, washability of components is important to the preservation of normal user patterns of care and maintenance of clothing.

In the torso integration, the raw pilot test data indicates that foam sensors can provide detectable responses to all of the body signals investigated, although careful sensor placement is important to the quality of data gathered. In this study, inter-subject anthropometric variation was controlled by limiting the number of subjects to one, and by custom-engineering the garment to fit that subject precisely. However, in a real-world scenario such control would not be possible, and sensor locations across a broad variety of body shapes and sizes would be hard to predict. Similar issues would arise with sizing, fit, and sensor locations on the foot. Because of the increased number of sensors and precision of locations, this variable would become even more difficult to control, however were the number and locations of sensors increased still more to create a uniform grid of pressure sensors, the fit issue could be avoided.

An additional problem of hysteresis caused by the PU foam substrate results in the gradual and positive increase in the resistance of the foam sensor. Since the position and the relative resistance of the PPy-coated PU sensors are crucial to their sensitivity, calibration of the sensors would be required on a regular basis. This calibration would involve setting the baseline resistance and range of the measured resistance of the sensors as determined through a series of standard repeatable exercises by the subject. Once these parameters are set subject monitoring could be commenced.

There are many applications of wearable sensing for which this type of sensor is particularly well suited. For example, in the monitoring of high-pressure body areas for individuals with reduced tactile sensation (such as diabetics suffering from neuropathy) the foam sensor would allow pressure points to be monitored without introducing a solid sensor element into a pressurized area close to the skin that could create more irritation. Rigid sensors in such an area could easily create more irritation and exacerbate the problem, but a foam sensor not only would not create irritation, it could actually protect the body from irritants by providing an additional layer of cushioning on key pressure points.

Outside of medical applications, knowledge of the state of the body is essential in many wearable, mobile, and ubiquitous computing applications. It is common in wearable and ubiquitous computing applications for a system to make decisions based on its perception of the needs and wants of the user. A subtle, comfortable sensor that demands no attention or adaptation from the user can allow the application to function invisibly, reducing the cognitive load on the user.

## Conclusion

Based on these preliminary data, polypyrrole-coated conductive foam shows considerable promise as a basic sensing technology, and for use in detecting body movements, physiological functions, and body state from body-garment interactions. Importantly, the sensor maintains the attractive structural properties of foam, consistent with the objectives of wearability and comfort in a smart garment.

Further study is necessary to fully understand the ability of the foam to serve as a reliable sensor over time and under the hostile conditions that garments must usually face. For instance, further work is required to understand and determine the effects of oxidation on baseline drift, the influence of variable conductance responses, calibrations of these responses and the optimal locations for sensors. In addition, processing algorithms for extraction of patterns from gathered data are required, as well as wearable and wireless hardware to allow the data to be used in real-time.

Future work includes in-depth analysis of foam responses in controlled environments, and evaluation of optimal sensor location for monitoring of specific activities and conditions.

## Competing interests

The author(s) declare that they have no competing interests.

## Authors' contributions

LED created the garment prototypes, participated in the prototype pilot evaluations, and drafted the manuscript. SB created the foam sensors, participated in the prototype pilot evaluations, and drafted the manuscript. BS participated in the project organization and supervised the research. DD participated in the project organization and supervised the research. All authors read and approved the final manuscript.

## References

[B1] De Rossi D, Carpi F, Lorussi F, Mazzoldi A, Paradiso R, Scilingo EP, Tognetti A (2003). Electroactive Fabrics and Wearable Biomonitoring Devices. AUTEX Research Journal.

[B2] Hertleer C, Grabowska M, Van Langenhove L, Catrysse M, Hermans B, Puers R, Kalmar A, van Egmond H, Matthys D Towards a Smart Suit. Proceedings of Wearable Electronic and Smart Textiles: Leeds, UK.

[B3] Malinauskas A (2001). Chemical depositing of conducting polymers. Poly.

[B4] Brady S, Diamond D, Lau KT Inherently conducting polymer modified polyurethane smart foam for pressure sensing,.

[B5] Thieblemon JC, Planche MF, Petrescu C, Bouvier JM, Bidan G (1993). Stability of Chemically Synthesized Polypyrrole films,. Synth Met.

